# Case Report: Nebulized platelet-rich plasma facilitates resolution of post-infective pulmonary consolidation and cavitation in a lung cancer survivor

**DOI:** 10.3389/fmed.2025.1738044

**Published:** 2026-01-13

**Authors:** Zane Sherif, Kirralee Sherif

**Affiliations:** Mermaid Beach Radiology, Gold Coast, QLD, Australia

**Keywords:** cavity, computational tomography, infection, lung, PRP, radiology, treatment

## Abstract

**Background:**

Persistent lung consolidation and cavitary lesions are recognized sequelae of severe pneumonia, particularly in patients with prior oncologic or radiation-related lung injury. Platelet-rich plasma (PRP) contains growth factors and cytokines that promote tissue repair and immune modulation, yet its pulmonary application remains largely unexplored.

**Case presentation:**

A woman in her mid-40s with a history of stage IV right-lower-lobe non-small-cell lung cancer (NSCLC) in long-term remission developed severe pneumonia while overseas. Six weeks after discharge, a low-dose CT revealed persistent right-upper-lobe consolidation with internal lucency. She administered nebulized autologous PRP for 4 weeks using a compressor-based nebulizer.

**Results:**

Serial CT imaging demonstrated rapid reduction and eventual resolution of the consolidation with closure of a cavitary defect. The patient experienced no bronchial irritation or adverse effects.

**Conclusion:**

Nebulized autologous PRP was temporally associated with radiologic resolution of a post-infective cavitary lesion in a previously irradiated lung. This finding supports further study of PRP's regenerative potential in pulmonary disease.

## Introduction

Persistent radiological abnormalities—including consolidation and cavitation—are well documented in the convalescence phase of severe pneumonia, particularly among patients with a history of cancer or thoracic radiotherapy (1, 2). The timeline for radiographic resolution may extend far beyond clinical recovery, often exceeding 6–12 weeks and complicating management decisions (3).

Autologous platelet-rich plasma (PRP) is widely used in regenerative medicine because of its high concentration of growth factors and anti-inflammatory cytokines (4). Although PRP has well documented clinical benefits in musculoskeletal and dermatologic its use in respiratory pathology remains poorly characterized. We describe a case in which nebulized PRP was associated with rapid radiologic improvement of a persistent post-infective pulmonary lesion in a cancer survivor.

## Case description

A woman in her mid-40s with prior stage IV right-lower-lobe NSCLC (diagnosed 2018; recurrence 2023) achieved complete remission following targeted systemic therapy, radiotherapy, and adjunct regenerative treatments. Her staging CT in May 2024 showed no evidence of active disease.

In October 2024, she developed a febrile lower-respiratory infection while overseas. After returning to Australia, she was hospitalized with presumed bacterial pneumonia and received six days of intravenous antibiotics. Although symptoms resolved, a low-dose follow-up CT performed 46 days later revealed a persistent right-upper-lobe consolidation with internal lucency, concerning for unresolved infection, abscess formation, or organizing pneumonia.

After informed consent, she commenced nebulized autologous PRP prepared from >60 ml of whole blood, producing a >10 × platelet concentration with high leukocyte content and no exogenous activation—corresponding to PAW classification P4-Aα (5, 6). Treatment was administered once daily for 4 days, then twice weekly for approximately 4 weeks.

### Imaging timeline

**Figure 1:**
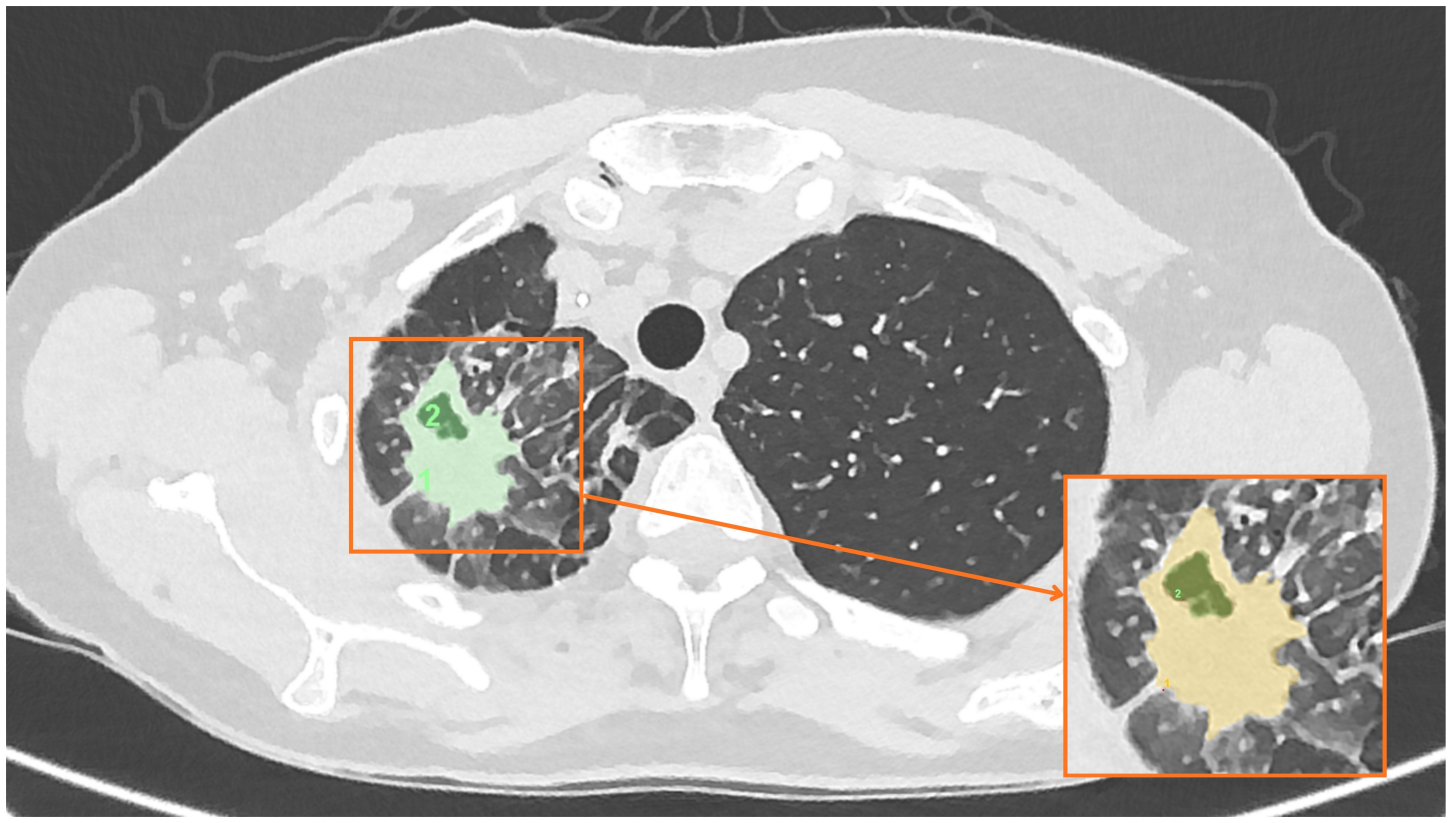
Baseline low-dose CT chest at Day 0 (initiation of nebulized PRP treatment) demonstrating persistent pulmonary consolidation without interval resolution.

**Figure 2:**
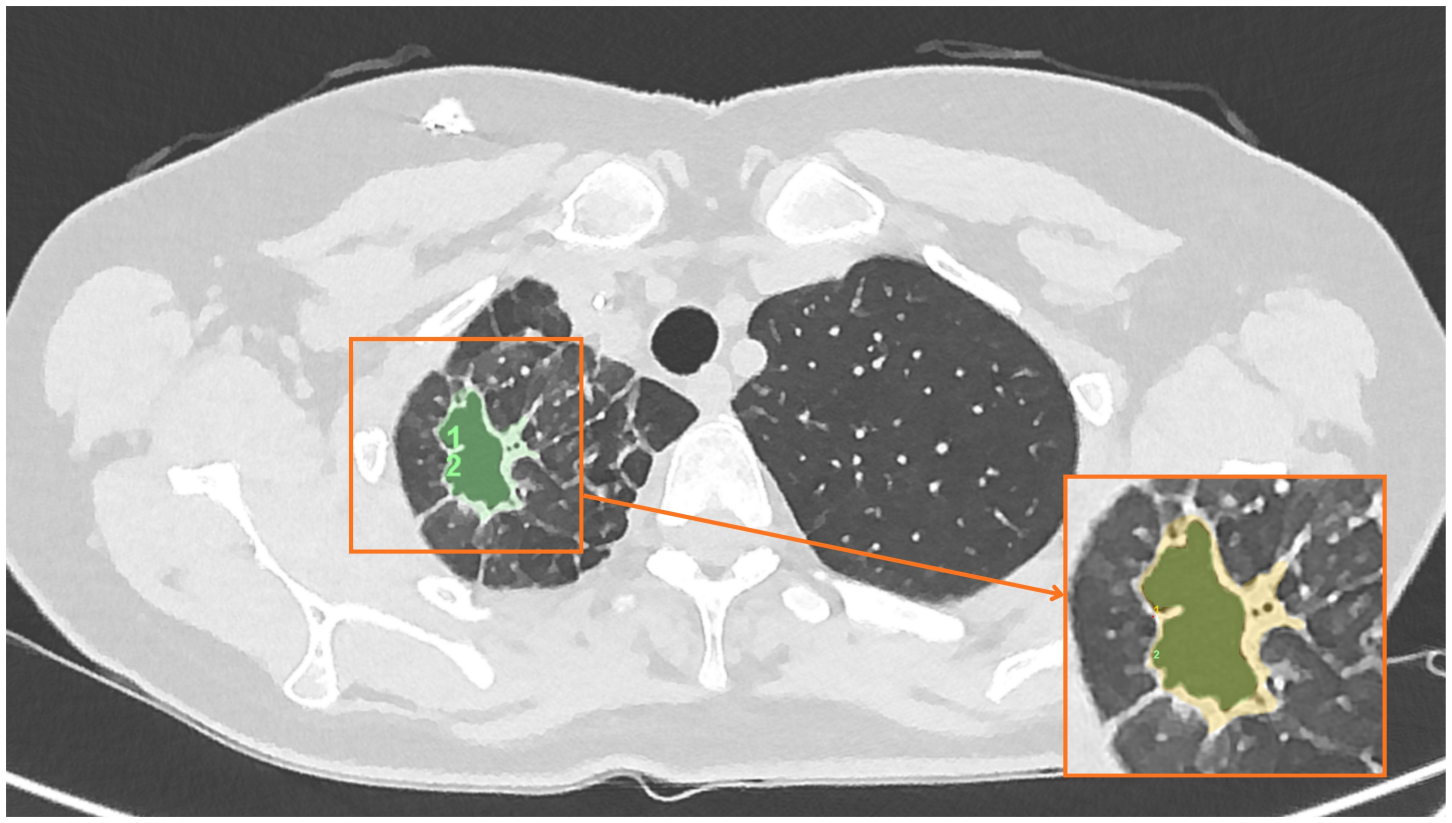
Low-dose CT chest at Day 15 post-treatment initiation demonstrating evolution to a cavitary component within the previously consolidated region, with surrounding parenchymal aeration consistent with interval organization and clearance.

**Figure 3:**
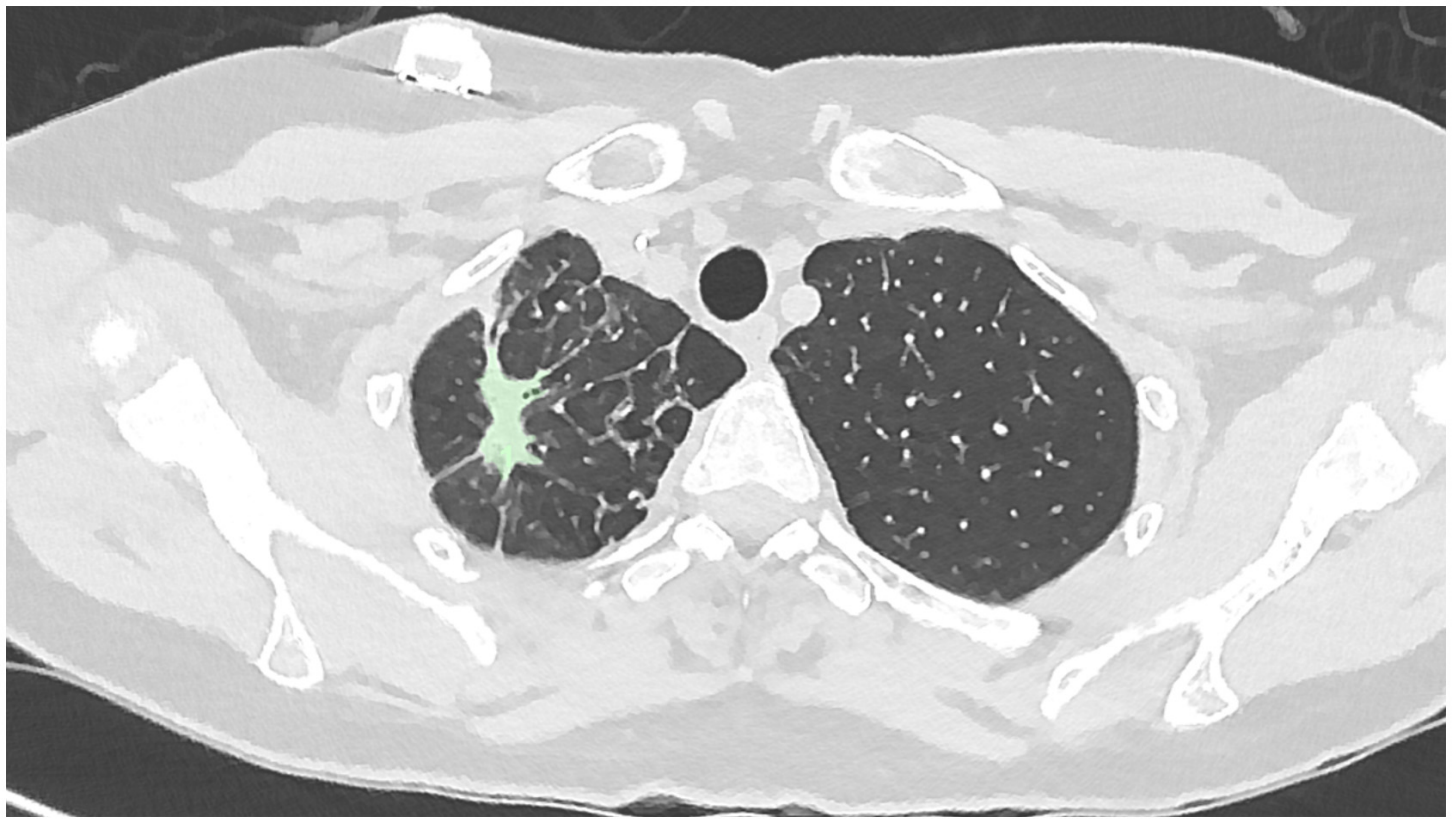
Low-dose CT chest at Day 64 post-treatment initiation demonstrating complete closure of the cavitary component with minimal residual parenchymal abnormality.

**Figure 4:**
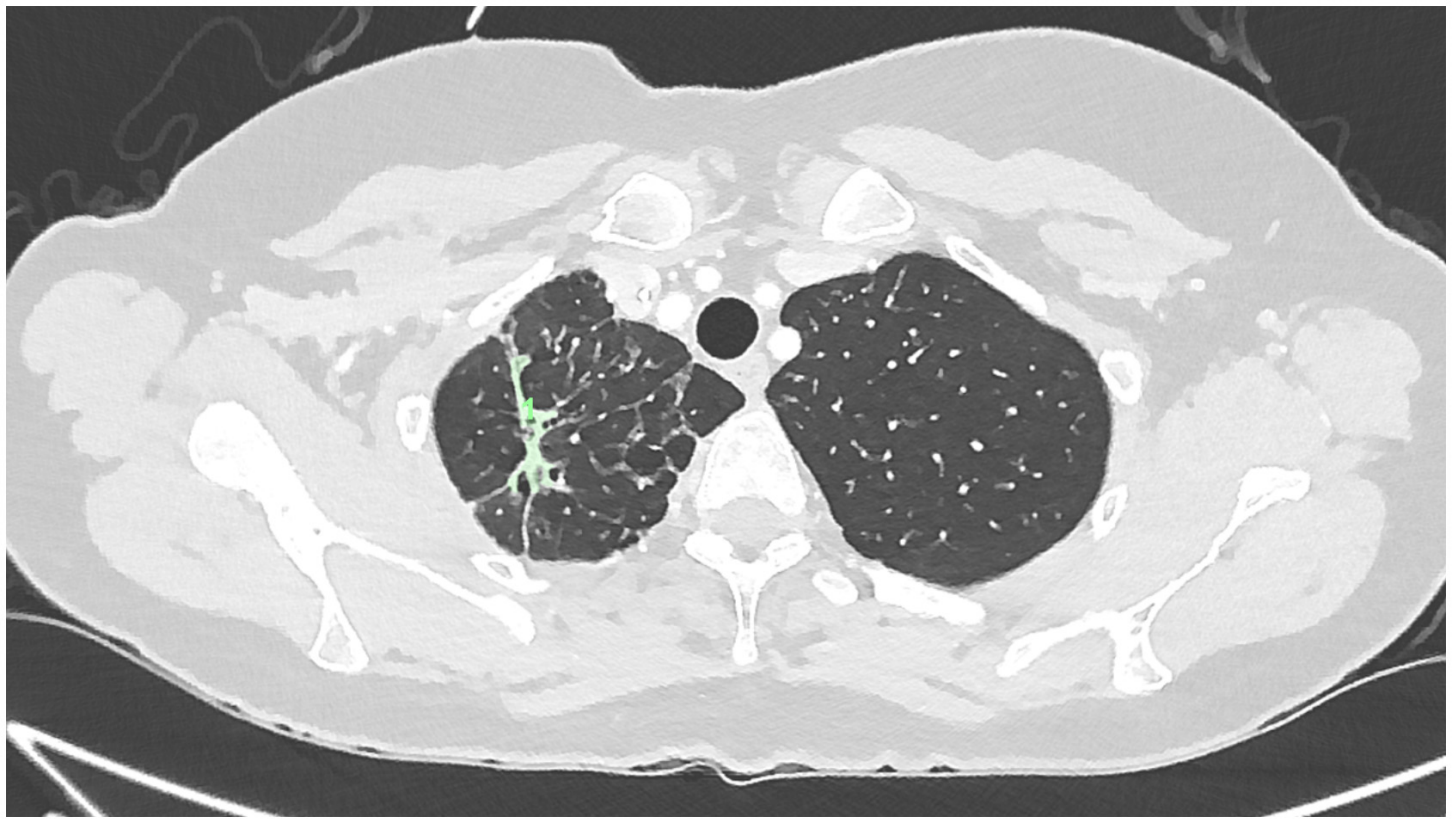
Low-dose CT chest at approximately Day 240 post-treatment initiation (8 months) demonstrating further reduction in residual scar tissue with stable parenchymal architecture.

The temporal evolution of radiological findings following initiation of nebulized autologous PRP treatment is summarized in Table 1, with representative low-dose CT images shown in Figures 1–4.

**Table 1:** Imaging timeline relative to initiation of nebulized PRP treatment.

**Figures**	**Time point (days post-treatment initiation)**	**Key radiological findings**
Figure 1	Day 0 (baseline)	Cavitating pulmonary consolidation without interval resolution at the time of PRP initiation. Dominant consolidation measured up to approximately 717 mm^2^ in cross sectional area.
Figure 2	Day 15	Evolution of a cavitary component within the previously consolidated region, with surrounding parenchymal aeration consistent with interval organization and clearance. Cavitary component extended across up to 512mm^2^ in cross sectional area.
Figure 3	Day 64	Complete closure of the cavitary component with minimal residual parenchymal abnormality
Figure 4	Day 240 (8 months)	Further reduction in residual scar tissue with stable parenchymal architecture

### Outcome and follow-up

The patient remained clinically stable with no wheeze, dyspnoea, or systemic symptoms. Serial imaging confirmed progressive resolution and full closure of the cavitary lesion 64 days after treatment. No biochemical or inflammatory derangements were observed.

## Discussion

Persistent post-infective pulmonary consolidation is frequently observed in patients with prior oncologic or radiation-related lung injury, likely due to impaired parenchymal healing mechanisms (1, 2). In a recent population-based study, Nguyen et al. reported that a history of lung cancer was independently associated with delayed radiologic resolution and a higher incidence of cavitary evolution following pneumonia (1). Earlier work by Macfarlane et al. demonstrated that radiographic abnormalities persist in up to 30% of adults at 12 weeks post-infection, even in the absence of ongoing clinical symptoms (3).

Autologous PRP contains a complex mixture of regenerative mediators, including platelet-derived growth factor (PDGF), vascular endothelial growth factor (VEGF), and transforming growth factor beta (TGF-β), which support angiogenesis, tissue remodeling, and epithelial repair (4). In this case, a leukocyte-rich PRP formulation was used despite theoretical concerns about bronchial irritation or cytokine-mediated inflammation. The patient experienced no adverse symptoms, and a mild productive cough may have contributed to mechanical clearance of necrotic material from the cavitary lesion.

On baseline imaging, the lesion demonstrated a maximal cross-sectional area of approximately 717 mm^2^, predominantly composed of dense consolidation, with a relatively small cavitary component measuring approximately 89 mm^2^. At 15 days following initiation of nebulized PRP therapy, the overall lesion cross-sectional area had reduced to approximately 512 mm^2^, with interval evolution to a cavitary-predominant morphology, the cavitary component measuring approximately 314 mm^2^. By day 64, the cavitary component had completely resolved, leaving residual linear parenchymal scarring measuring approximately 257 mm^2^, which further decreased to approximately 166 mm^2^ at the 8-month follow-up.

This case report is limited by its radiology-led, observational design. The patient was clinically asymptomatic at the time of imaging assessment and was not under the care of a respiratory physician. No pulmonary function tests, arterial blood gases, or inflammatory biomarkers (e.g., CRP, procalcitonin) were obtained, as there were no respiratory symptoms or clinical indications for further physiological evaluation. The findings reported in this case therefore reflect radiological evolution rather than clinical outcome measures.

Nebulized drug delivery offers several advantages in pulmonary medicine, including high local deposition with minimal systemic absorption. This method has shown promise in other inflammatory respiratory conditions, such as COVID-19 pneumonia, where aerosolized anti-inflammatory agents including budesonide and N-acetylcysteine (NAC) improved clinical and radiologic outcomes (7, 8). The same delivery principles may be applicable to autologous biologics such as PRP, particularly when targeting focal airway or parenchymal abnormalities.

Pre-clinical studies further support the biological plausibility of inhaled platelet-derived therapeutics. Du et al. (9) demonstrated that nebulized platelet-derived extracellular vesicles significantly attenuated emphysematous lung injury in murine models, while Rizzo et al. (10) confirmed preferential pulmonary biodistribution of platelet-derived regenerative exosomes following aerosol delivery in a large-animal model. Although PRP differs compositionally from isolated vesicles, these findings provide mechanistic support for localized platelet-mediated repair within injured lung parenchyma.

Although causality cannot be definitively determined from a single case, the consistent anatomical response and temporal proximity between PRP administration and consolidation and cavity resolution suggest a possible therapeutic effect.

## Patient perspective

After being discharged, I felt mostly recovered—but the scan was shocking, especially given my cancer history. I didn't want more antibiotics. The nebulized PRP was easy to use, and seeing the improvement on the scan was amazing.

## Data Availability

The original contributions presented in the study are included in the article/supplementary material, further inquiries can be directed to the corresponding author.
